# Anti-Weightlessness Physiological Protection for the Lower Limb Muscle System Based on Biomimetic Adhesive Force Stimulation

**DOI:** 10.3390/biomimetics10120800

**Published:** 2025-11-28

**Authors:** Yuanming Ji, Zhili Li, Peng Zou, Chengyang Li, Xipeng Wang, Xiyue Yang, Zhendong Dai, Keju Ji

**Affiliations:** 1 Jiangsu Provincial Key Laboratory of Biomimetic Materials and Devices, Nanjing University of Aeronautics and Astronautics, Nanjing 210016, China; jiyuanming@nuaa.edu.cn (Y.J.); lichengyang@nuaa.edu.cn (C.L.); wxp114@nuaa.edu.cn (X.W.); zddai@nuaa.edu.cn (Z.D.); 2 State Key Laboratory of Space Medicine, China Astronaut Research and Training Center, Beijing 100094, China; 3 Electric and Information Engineering, Electric and Electronic Engineering, Imperial College London, South Kensington Campus, London SW7 2AZ, UK; aryayang1017729@163.com

**Keywords:** biomimetic adhesion, multi-level hierarchy, microstructure, microgravity countermeasure, van der Waals force, electromyography (EMG) signals

## Abstract

With the advancement of crewed spaceflight, mitigating the physiological effects of microgravity, such as bone–muscle deterioration and movement instability, has become increasingly vital. Inspired by reptilian climbing mechanisms, this study presents a novel bio-inspired adhesive footwear characterized by low pre-load, strong adhesion, and controllable attachment–detachment capability. This study analyzes the adaptability of a multi-level variable modulus design to surfaces with varying curvatures and roughness. Experimental investigations were conducted to analyze the contact mechanics and interfacial mechanisms of biomimetic adhesive materials featuring microstructure arrays. Moreover, stepping exercises were performed by volunteers wearing the proposed footwear under simulated weightlessness to assess biomechanical performance. Interface contact stresses were measured using force-sensing array plates, enabling characterization of plantar adhesion under different detachment speeds and angles. Electromyographic signals from lower limb muscle groups during stepping exercises were analyzed to elucidate the mechanical stimulation patterns and effects induced by plantar adhesion forces. Results indicate that plantar adhesion forces ranging between 50 and 105 N effectively stimulate primary flexor muscles, including the biceps femoris and gastrocnemius. This biomimetic solution offers a flexible and convenient approach for stabilizing foot positioning and promoting musculoskeletal engagement in microgravity, improving astronauts’ mobility and operational performance in orbit.

## 1. Introduction

Space exploration represents the frontier of human understanding and the transformation of nature, rendering it a pursuit of paramount importance. The development of crewed space technology directly reflects a nation’s comprehensive strength and enhances its international influence. Although modern spacecraft are equipped with advanced automated and intelligent systems capable of autonomously performing various tasks, such as observation, data collection, and processing, human involvement remains indispensable. Astronauts’ on-site judgment, emergency response capabilities, and adaptability to complex situations are critical for mission success. Therefore, astronauts constitute the most valuable resource in space exploration and drive the continuous advancement of human space endeavors.

In outer space, astronauts encounter challenges in daily life and work that differ drastically from those on Earth. In microgravity, foot muscles no longer support the body’s weight, requiring astronauts to adjust their posture and movement to maintain balance. This adjustment requires continuous exertion of specific muscle groups. Prolonged maintenance of these postures impacts work efficiency and damages the muscular system’s structure and function. In most simulated weightless environments, such as those created by suspension systems or air-cushion beds, the maximum plantar force experienced by astronauts is reduced by approximately 70–82% compared to the gravitational force on Earth, with the vertical impulse decreasing by 35–52% [1,2,3,4,5]. Although gait cycle times do not significantly increase compared to those in normal gravity conditions, the stance phase, which plays a crucial role, is significantly shortened. The notable reductions in maximum plantar force, stance duration, and vertical impulse indicate diminished mechanical stimulation to the lower limb muscular system, leading to muscle atrophy. Consequently, astronauts often experience impaired musculoskeletal function upon returning to Earth after extended space missions. Additionally, the plantar surface cannot generate a significant frictional cone with contact surfaces without gravity, causing astronauts to float unrestrained in microgravity. Therefore, enabling astronauts to achieve flexible and reliable position fixation, walking, and exercise in microgravity, resembling their movement on Earth, remains a primary focus in microgravity countermeasure research.

Existing exercise methods, including treadmill training, resistance training, and cycling [6,7], support the countermeasure needs in weightless environments. However, these devices are bulky and do not provide comprehensive musculoskeletal training, which can still affect muscle function. Recent studies have shown that during running exercises in space [8,9,10,11], the maximum plantar force is only 54% of that on Earth, insufficient to adequately stimulate lower limb muscles and prevent muscle degradation [12]. The existing foot fixation method using restraint bands [13] relies on a cooperative fixation approach that lacks flexibility and can cause fatigue or injury due to prolonged dorsal foot pressure. Consequently, astronauts often experience difficulty readapting to Earth’s gravity upon completion of long-term space missions.

Among the existing fixation methods, magnetic adhesive nylon hook-and-loop fasteners [14] require compatible surfaces for effective adhesion, limiting astronaut mobility and task performance. Moreover, the magnetic fields generated by magnetic adhesion often interfere with onboard instruments, risking performance degradation, malfunction, or even damage. Claw-like attachments [15], although enabling fixation, are only effective on rough surfaces, restricting their applicability. Therefore, an effective device is required that can support astronauts’ positional stability, walking, and exercise.

Animals such as geckos, flies, and spiders rely on the van der Waals forces between their feet and the target surfaces to achieve omnidirectional climbing ability. The attachment ability of these animals [16,17,18,19] is due to their foot adhesion functional units, which feature arrays of setae combined with multi-level adaptive structures, enabling reliable contact with the target surface. In 2014, the Russian space agency sent a gecko into space [20,21,22,23]; the gecko successfully maintained stable interface attachment and movement within the microgravity environment of the space station, demonstrating the effectiveness and adaptability of van der Waals-based mechanisms. The ability to flexibly attach to and climb vertical and ceiling surfaces in space can address astronauts’ needs for secure surface fixation and enhanced stability in microgravity environments, providing a biological blueprint for assistive devices. This could enable astronauts to achieve omnidirectional mobility similar to that of climbing organisms while operating in space.

This paper presents a biomimetic adhesive shoe that integrates an array of microstructured adhesive units in its sole, composed of a biomimetic adhesion layer, a cushioning adaptive layer, and an upper wearable portion. Experiments were conducted to investigate and analyze the adhesive mechanical properties and underlying mechanics of the biomimetic adhesive material. Interface contact mechanics were evaluated under various surface conditions and attachment–detachment parameters (detachment speed, angle, preload, and substrate roughness). The feasibility of the biomimetic adhesive shoe as an assistive tool for lower limb musculoskeletal training was validated by stepping exercises performed in simulated microgravity conditions

## 2. Experimental Section

### 2.1. Fabrication of Biomimetic Adhesive Shoe

The biomimetic adhesive shoe comprises a biomimetic adhesion layer, a cushioning adaptive layer, and an upper wearable portion. The biomimetic adhesion layer was fabricated using a biomimetic adhesive material featuring an array of microstructures. Specifically, the photoresist was exposed and developed by a photolithography machine (Suss MA-6) using the photolithography process, and a mold with a microstructure array was obtained. Then, the prepolymer silica gel (No. 4130) was prepared using glue injection equipment at a rate of 2 mL/s, 0.4 MPa pressure into the microstructure mold to pour molding, which was then heated and solidified at 70 °C for 10 h; then, the mold was released to obtain the bionic adhesive layer. A porous nylon felt material (primary cushioning adaptive layer) with a tensile strength of 3.2 MPa and a tear strength of 1.3 MPa was used as a backing substrate to ensure robust bonding with the cushioning adaptive layer. The nylon felt and silicone prepolymer were simultaneously introduced during the molding process, enabling integral fabrication of the nylon felt with the biomimetic adhesion layer.

To further improve the performance of the cushioning adaptive layer, we fabricated a secondary adaptive layer comprising a micropillar array structure using a combination of 3D printing and molding techniques. This secondary adaptive layer was made from 50 A rubber and thermally bonded to the backside of the nylon felt. The adhesive sole of the biomimetic shoe was designed as discrete, three-segment adhesive units: forefoot, midfoot, and rearfoot regions. Considering the shape characteristics of plantar contact areas, circular contact zones were designated in the forefoot and rearfoot sections, ensuring that stress-concentrated regions during walking and stepping are effectively supported, thus dispersing localized stresses and enhancing comfort.

The upper wearable portion was crafted from cowhide leather and attached to the secondary cushioning adaptive layer using a combination of nylon-thread stitching and adhesive bonding. The final biomimetic adhesive shoe structure, from bottom to top, is as follows: the biomimetic adhesion layer, primary cushioning adaptive layer, secondary cushioning adaptive layer, and upper wearable portion.

### 2.2. Contact Mechanics and Exercise Efficacy Testing of the Biomimetic Adhesive Shoe

The experimental evaluation comprised tests on the biomimetic adhesive units and the adhesive shoe as a whole. The biomimetic adhesive unit with buffering and adhesion functions was labeled BA (Buffer and Adhesion), and its corresponding shoe was labeled BA-S (Buffer and Adhesion Shoes). The control groups included adhesive units and shoes without buffering layers, labeled N-B (No-Buffer) and N-B-S (No-Buffer Shoes), respectively. Additionally, adhesive units and shoes with buffering but without microstructured biomimetic adhesion layers were designated N-A (No-Adhesion) and N-A-S (No-Adhesion Shoes), respectively.

#### 2.2.1. Contact Mechanics Testing of Biomimetic Adhesive Units

Normal adhesion forces were measured using a Bruker UMT tribometer (Saarbr ü cken, Germany Bruker). Samples of dimension 1 cm × 1 cm were prepared by precision cutting with a vibration knife, mounted onto the upper fixture using double-sided tape, while substrates of glass (surface roughness Sa = 5 µm) and aluminum alloy plate (Sa = 0.2 µm) were fixed onto the lower fixture. A preload of 5 N and a detachment speed of 1 mm/s were applied, and adhesion force changes over time were recorded. Tangential adhesion forces were evaluated on an aluminum alloy substrate using a self-developed 3D force sensor setup. Surface roughness levels (Sa = 0.02–5 μm) were verified by laser confocal microscopy; representative roughness maps and parameters are shown in Figure S1. A string attached to the edge of the biomimetic adhesive unit was subjected to increasing tangential pull until detachment from the substrate occurred.

The fatigue life of the biomimetic adhesive shoes was assessed using a rubber fatigue testing machine. Contact mechanics and surface morphology changes before and after fatigue testing were compared to evaluate durability and reusability.

#### 2.2.2. Adhesion Force Testing of Biomimetic Adhesive Shoes

Adhesion performance under simulated microgravity conditions was tested using a self-developed microgravity simulation platform integrated with a force measurement platform. A constant-tension suspension system was used to emulate reduced effective body weight; the device layout is shown in Figure S2, and the statics/geometry leading to the approximately constant tensile support are detailed in Supplementary Methods S3.1 (Equations (S1)–(S4)). Ten volunteers (age: 20–35 years, mean age: 26 years; five males, five females) participated in the study. Each participant was suspended vertically using a constant-force suspension system to counterbalance body weight, thereby simulating microgravity. An aluminum alloy plate with a matte finish (roughness 0.2 µm) was mounted on the force platform. Participants performed adhesion and detachment actions between the shoe sole and aluminum alloy plate under five different detachment modes, with real-time interface contact mechanics data recorded by the platform.

Detachment methods included: vertical detachment, detachment from forefoot to rearfoot, detachment from rearfoot to forefoot, medial-to-lateral detachment, and lateral-to-medial detachment. Detachment speeds were controlled by varying stepping frequencies regulated by a metronome, enabling assessment of interface mechanics at different detachment velocities. Adhesion performance was further evaluated on substrates of varying roughness to characterize adaptability to diverse surface conditions.

#### 2.2.3. Electromyography (EMG) Signal Testing

Surface EMG signals were acquired using the BTS FREEEMG 300 wireless electromyography system. Surface electrodes were placed on the major muscle groups of the lower limbs. Volunteers were first suspended in a simulated microgravity environment by the constant-force suspension device, performing stepping exercises without the biomimetic adhesive shoes for 5 min as a baseline control test. Subsequently, the tests were repeated with the biomimetic adhesive shoes. EMG signals from selected muscle groups were continuously recorded throughout the tests. Raw EMG signals were processed in MATLAB R2023b (9.16.0) 64-bit (MathWorks, Natick, MA, USA) using a 4th-order Butterworth bandpass filter to remove noise outside the frequency range of 20–400 Hz, followed by a root mean squared (RMS) smoothing procedure with a window size of 50 data points to obtain RMS-processed EMG values. Surface electrode locations for BF, TA, LG, GM, and VL followed standard guidelines; a placement map is provided in Figure S3 (Supplementary Materials). All anonymized paired observations (per subject and condition; loads, *EMG_RMS_*, Δ*EMG%*, and trial indices) are available in Dataset S1.

## 3. Results and Discussion

**Figure 1:**
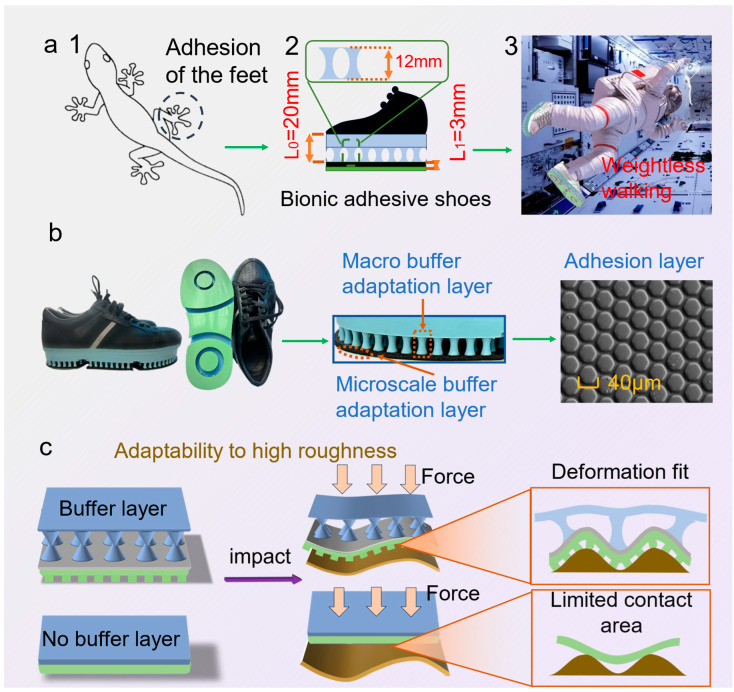
(**a**) (**1**) The gecko’s foot morphology during climbing, (**2**) the structure of the biomimetic adhesive shoe inspired by this mechanism, and (**3**) simulated walking in a weightless space environment using the biomimetic adhesive shoe; (**b**) side-top view of biomimetic adhesive shoe, small local enlarged view, and topography of adhesive microstructure arrays; (**c**) schematic diagram of the adaptability of the multi-level buffer structure to the surface and roughness.

Inspired by the hierarchical setal array of gecko toes, the biomimetic adhesive shoe integrates multi-level, cross-scale cushioning adaptive layers with a microstructured adhesive layer. L_0_ denotes the characteristic length/compliance-path scale of the macro-buffer layer, governing energy dissipation in the load–deformation process and the redistribution of interfacial loads; L_1_ denotes the characteristic microstructural contact length, primarily controlling the evolution of real contact area and the efficiency of adhesive-energy utilization. This design aims to provide astronauts with an efficient fixation and walking method based on biomimetic adhesion technology under microgravity conditions (Figure 1a). Following a Buffer→load-shaping→Adhesion (BA) chain, L_0_ facilitates pressure equalization and peak attenuation, whereas L_1_ affords van der Waals–dominated, direction-selective traction with reversible release under modest preload and a small tangential bias; key geometric/operating parameters (L_0_, L_1_, $F_{N}$, peeling angle α) are annotated in Figure 1a to enable direct mapping from biology to engineering. Figure 1b illustrates the morphology of the biomimetic adhesive shoe sole, highlighting the multi-level cushioning adaptive layers and an outer biomimetic adhesive layer. The adhesive layer comprises a hexagonal prism array with an average circums cribed diameter of approximately 40 μm, an average height of 15 μm, and an average gap of 4 μm. The prisms are arranged in an equidistant pattern, with a center-to-center spacing of 45 μm between adjacent prisms.

The macro cushioning adaptive layers significantly enhance adaptability to adhesive surfaces with varying curvature and friction coefficients. Specifically, the macro-scale cushioning layer adapts to the target surface curvature through substantial deformation under moderate preloads, while the microstructured adhesive layer further accommodates surface roughness, increasing real interfacial contact at the microscale and effectively adapting to substrates of different roughness (Figure 1c).

### 3.1. Contact Mechanics Behavior of Biomimetic Adhesive Units

**Figure 2:**
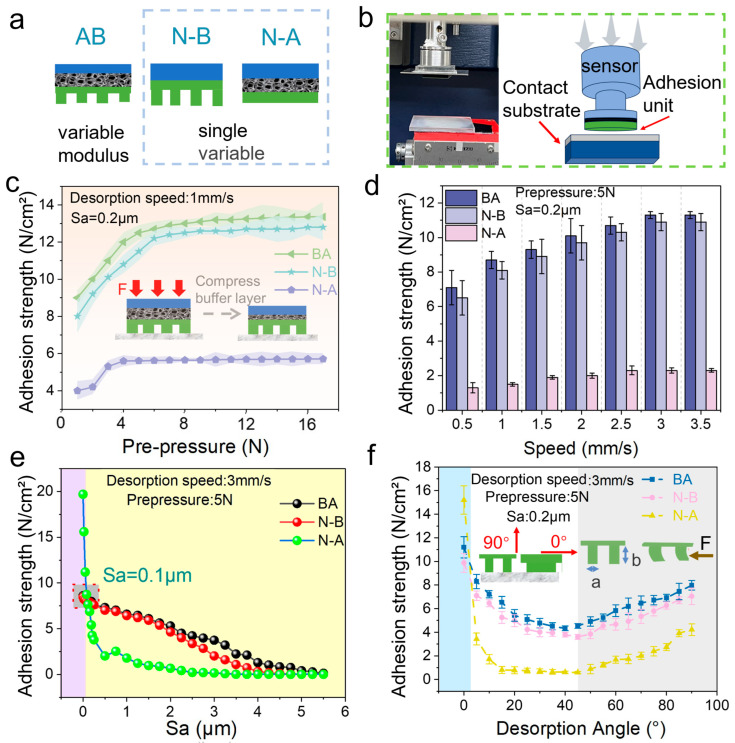
(**a**) Samples with buffer layer and microstructure layer (AB), samples with buffer layer and microstructure layer (N-B), and samples with buffer layer and no microstructure layer (N-A); (**b**) physical drawings and schematic diagrams of the experimental apparatus for testing biomimetic adhesive materials; (**c**) adhesion of the sample under different pre-pressures; (**d**) adhesion of the sample at different detachment speeds; (**e**) adhesion of the sample on substrates with different roughness; (**f**) adhesion of the sample at different angles, with insets showing the detachment direction and pillar height.

The contact mechanical properties of biomimetic adhesive units on various target surfaces are presented in Figure 2. Figure 2a presents a schematic diagram of the biomimetic adhesive unit (BA) and comparative structural units (N-B and N-A). The experimental apparatus and testing methods used to evaluate contact mechanical behavior are illustrated in Figure 2b.

Figure 2c displays the variation in normal adhesion forces with preload ranging from 0.1 N to 8 N, at a surface roughness of 0.2 μm and detachment speed of 3 mm/s. The BA unit exhibits a significant increase in adhesion force (over tenfold), demonstrating effective adhesion and stable attachment at relatively low preload. Conversely, the adhesion force of the N-A unit is substantially lower owing to the absence of microstructures, resulting in reduced adaptability and contact area. For the BA unit, adhesion force plateaus beyond a preload of approximately 5 N, indicating that the microstructural elements achieve near-complete molecular-level contact, and additional preload yields minimal gains. The N-B unit requires a higher preload peak compared to the BA unit, as the buffer layer in the BA structure more evenly distributes stress, enabling complete contact at lower preload values (Figure 2c inset).

Figure 2d shows the adhesion force of the biomimetic adhesive units at different detachment speeds, with a fixed preload of 5 N and substrate roughness (Sa) of 0.2 μm. The adhesion force increases from approximately 7 N at 0.5 mm/s to approximately 11 N at 3 mm/s. Beyond 3 mm/s, adhesion force plateaus, indicating that the molecular adhesion formation rate has reached its limit. According to the momentum theorem (ft = mv), higher detachment speeds reduce detachment time and thus require greater force. The BA group exhibits higher adhesion than the other groups owing to the increased microscopic contact area facilitated by its microstructured adhesive layer. The lack of a buffer layer in N-B causes uneven stress distribution and a slight reduction (approximately 5%) in adhesion. The N-A group shows the lowest adhesion owing to its minimal contact area.

Figure 2e presents the adhesion performance of units tested on acrylic substrates with varying roughness levels (detachment speed of 3 mm/s, preload of 5 N). Corresponding roughness maps for the tested substrates are provided in Figure S1, which corroborate the monotonic decrease in adhesion with increasing Sa. Adhesion force decreases linearly with increasing surface roughness, as higher roughness reduces the microscopic contact area. Notably, at very low roughness (Sa < 0.1 μm), N-A exhibits significantly higher adhesion (over 250%) due to its larger effective contact area on smoother surfaces. However, BA outperforms N-B at higher roughness values owing to its buffering layer, enabling better conformity and contact. Adhesion significantly decreases from Sa = 2.0 μm to Sa = 3.5 μm, and nearly vanishes at Sa = 5.0 μm due to reduced molecular contact probability.

Figure 2f illustrates the relationship between adhesion force and detachment angle for the BA unit, measured using a custom-developed 3D force-sensing platform (detachment speed: 3 mm/s, preload: 5 N, substrate roughness: Sa = 0.2 μm). The adhesion force of the BA unit initially decreases from 8.7 N at 0° to 4.3 N at 45°, before increasing again to 7.6 N at 90°. The N-B unit experiences uneven tangential force distribution, resulting in lower detachment force and noticeable stick-slip behavior. Conversely, BA and N-A units demonstrate smoother detachment. At angles less than 3°, the adhesion of N-A is significantly higher (>30%) owing to the frictional contribution from its larger contact area. Beyond 3°, the adhesion decreases sharply. The inset on the left side of Figure 2f illustrates the direction of tensile force with or without adhesive layers. Beyond 45°, the adhesion force primarily arises from normal adhesion; hence, the BA unit performs better. The right inset of Figure 2f shows the dimensional relationship between pillar height (b) and diameter (a). With a height greater than the diameter, micropillars bend under angled tension, maintaining sufficient contact area and mitigating adhesion loss caused by angle variation.

Fatigue testing under repeated normal adhesion–detachment cycles (10,000 cycles) revealed no significant reduction in normal adhesion force, with surface morphology exhibiting only minor micro-cracks. This confirmed the long-term stability and applicability of the biomimetic adhesive units for engineering applications.

### 3.2. Contact Mechanics Behavior of Biomimetic Adhesive Shoes Under Simulated Microgravity

**Figure 3:**
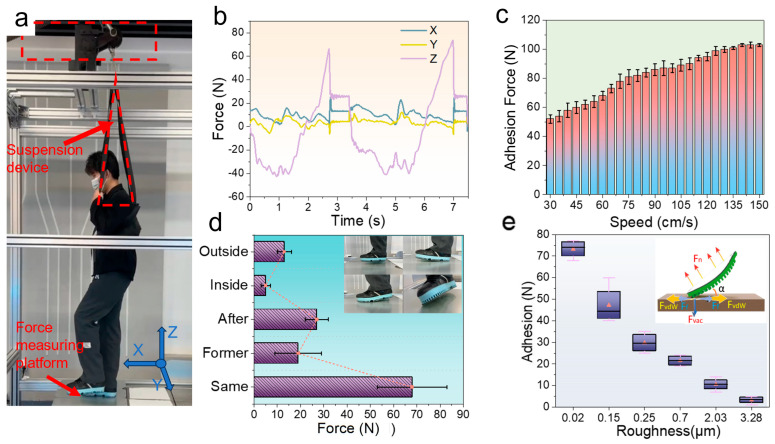
(**a**) Attitude diagram of the subject under the force measurement platform simulating a microgravity environment; (**b**) F_X_, F_Y_, and F_Z_ process data diagrams collected by the three-dimensional force measurement platform in the microgravity environment; (**c**) variation in adhesion of biomimetic adhesive shoes with different detachment speeds; (**d**) adhesion of the forefoot mode, hind heel mode, medial adhesion–detachment mode, and lateral detachment mode in microgravity, and the inset shows different detachment behavior patterns; (**e**) variation in the adhesion of biomimetic adhesive shoes under different roughness surfaces, and the inset shows a schematic diagram of microscopic detachment. A short demonstration of the suspension-based stepping tasks under the BA, N-A, and No-shoe conditions is provided in Video S1 (Supplementary Materials).

Participants wore biomimetic adhesive shoes and were suspended using a constant-force suspension system to simulate microgravity conditions (Figure 3a). Figure 3b illustrates the variations in plantar force recorded while participants stepped on a force-measurement platform, with stepping frequency controlled at 30 steps/min. At initial foot contact, the support force increased, peaking at approximately 100 N during the late stance phase. Subsequently, as the foot was raised, maximum plantar adhesion occurred before transitioning into the swing phase. Compared with a gravity-based load of approximately 600 N, this represents an approximately 85% reduction in force, confirming the effectiveness of microgravity simulation. The maximum normal adhesion force under simulated microgravity conditions was 75 ± 12 N, reflecting differences due to varying preload conditions during the stepping process.

Under vertical detachment conditions in a gravity environment, the adhesion performance was tested by participants lifting their feet to the same height (10 cm) at detachment speeds ranging from 30 cm/s to 150 cm/s (Figure 3c). Adhesion force increased with increasing detachment speed, with the peak force at 150 cm/s nearly double that at 30 cm/s. Additionally, the duration of force application decreased as speed increased. The detachment time at 30 cm/s was approximately five times longer than at 150 cm/s. Consequently, the adhesion force impulse decreased with increasing speed up to a certain threshold. Beyond approximately 110 cm/s, additional increases in adhesion force were minimal, indicating a limit in maximum achievable impulse given a fixed contact area.

Stepping exercises under simulated microgravity using different detachment modes (Figure 3d inset) revealed that the vertical detachment (simultaneous detachment) produced the highest adhesion force compared to the other four modes. The average adhesion force under simultaneous detachment was 67 ± 15 N (Figure 3d). This result occurs primarily because simultaneous stepping results in the heel and forefoot leaving the ground simultaneously, causing nearly all adhesive micropillars to detach simultaneously. Consequently, adhesion force predominantly acts in the normal direction, minimizing tangential friction and maximizing the observed adhesion.

Compared to other detachment modes, lateral and medial detachment modes are significantly influenced by tangential friction forces. Given that the total adhesion force remains approximately constant, normal adhesion forces under these modes are substantially lower than those observed in vertical and rear-side detachment modes. Specifically, the average normal adhesion force in lateral detachment was only 18.5% and in medial detachment, 28.3% of that observed in vertical detachment. This disparity arises from the differing microstructural interactions during lateral or medial detachment. Assuming the adhesive units’ micropillars contact a locally flat substrate, the adhesive interaction force (*P_f_*) between the shoe sole and the surface [24,25] is given by

(1) $$P_{f}=6\pi r3K\omega_{f}$$

(2) $$\omega_{f}=\gamma L(1+cos\theta )$$

where *P_f_* represents the normal adhesion force (N), *f* denotes the effective adhesion energy (mJ/m^2^), r is the radius of the micropillars (μm), K is the effective Young’s modulus (MPa), and *ω_f_* is the work of adhesion (W). γL represents liquid surface tension (N), and *θ* denotes the adhesion angle (°). According to Equations (1) and (2), the effective adhesion energy of the biomimetic adhesive shoe is influenced by the adhesion angle *θ*. During medial-side detachment, the inclination angle of the primary tensile force exerted on the lower leg relative to the *z*-axis ranges between 20° and 30°, with lateral forces on the coronal plane dominating the interaction. Consequently, approximately 30% of the adhesion force acts in the tangential direction and approximately 70% in the normal direction under gravitational conditions. As part of the tangential force is offset by interfacial stresses, detachment can be achieved using relatively smaller normal forces.

However, the maximum adhesion force under the forefoot detachment mode is only 8.9% of that observed during vertical detachment under identical conditions, considerably lower than other detachment modes. This significant reduction occurs because lifting the foot from the forefoot side creates a larger peeling angle approaching 90° between the forefoot area and the contact surface. A larger peeling angle facilitates crack propagation at the interface between the adhesive material and the substrate, enabling complete detachment with lower adhesion force. Conversely, the peeling angles in other detachment modes are typically smaller, rendering crack propagation more difficult and thus requiring higher forces to achieve detachment.

Surface roughness is a critical factor influencing the adhesion performance of the biomimetic adhesive shoes, particularly as contact surfaces in spacecraft exhibit varied roughness levels. Consequently, the biomimetic adhesive shoe must provide sufficient adhesion force while adapting effectively to different surface roughness conditions. The adhesion mechanism of biomimetic adhesive materials is illustrated schematically in the inset of Figure 3e. When micropillars are angled and pulled, both tangential friction forces and normal adhesion forces are generated, collectively ensuring adequate adhesion at certain inclination angles. For the biomimetic adhesive shoe with a multi-level hierarchical design under normal force, the frictional mechanism relies on dual effects: mechanical interlocking and van der Waals forces. Mechanical interlocking refers to the tangential resistance generated as the elastomer deforms and conforms to the microstructure arrays of the acrylic surface under normal force. As surface roughness increases, the effective interlocking area initially increases but decreases at higher roughness. Tangential adhesion forces resulting from van der Waals interactions can be expressed by Equation (3) [26,27,28]:

(3) $$F_{c}\sim \sqrt{G_{c}}\sqrt{A/C}$$

where *G_c_* represents the critical strain energy for interface separation, a constant for specific material interfaces. *A* denotes the actual contact area between the elastomer and the acrylic substrate, while *C* is the compliance of the elastomer sample in the direction of the frictional force. Increasing the interfacial contact area and decreasing the elastomer’s compliance enhances the tangential adhesion force. The variable-modulus biomimetic adhesive units address the interface properties required for high adhesion forces. The high-modulus backing layer of the adhesive unit reduces the elastomer’s tangential compliance, while the low-modulus contact layer increases compliance with surface roughness, thereby improving the actual contact area between the adhesive unit and the acrylic substrate. This results in a higher tangential adhesion force for the variable-modulus biomimetic adhesive unit.

As the surface roughness of the acrylic substrate increases from Sa = 0.02 μm to Sa = 3.3 μm, the adhesion force of the biomimetic adhesive shoes, tested under vertical detachment in a gravity environment, gradually decreases. A metronome was used to control the stepping frequency to 30 steps/min. Figure 3e shows that as the roughness increases, the adhesion force of the shoe decreases. At the microscale, higher roughness leads to fewer contact points between the biomimetic adhesive protrusions and the substrate, resulting in variations in the total adhesion force due to differences in the number of protrusions in contact with surfaces of varying roughness. Therefore, the performance of the biomimetic adhesive shoes can be estimated based on different levels of frictional forces.

In conclusion, increasing the contact area and reducing the compliance of the elastomer in the direction of the frictional force can significantly enhance the tangential adhesion force. The variable-modulus biomimetic adhesive unit optimizes this mechanism for effective performance across various surface conditions.

**Figure 4:**
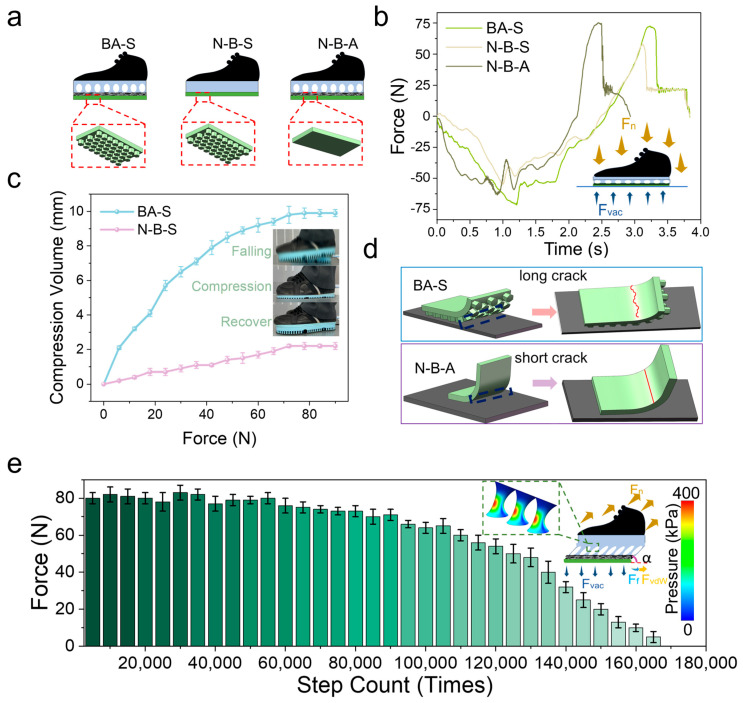
(**a**) Shoes with buffer layer and microstructure layer (BA-S), shoes with no buffer layer (N-B-S), and shoes with buffer layer and no microstructure layer (N-B-A); (**b**) mechanical cushioning responses of the three shoe types under a single impact, with the inset presenting the cushioning mechanics of the biomimetic adhesive shoes upon impact; (**c**) compression cushioning performance of BAS and N-B-S under varying impact conditions, with the inset showing the morphological evolution of the cushioning layer in BAS under a single impact; (**d**) crack propagation behavior of BAS and N-B-A during edge tearing; (**e**) variation in the adhesion force of BAS with increasing step cycles, with the inset illustrating the mechanical response of BAS under tensile loading and the corresponding stress distribution within the cushioning layer; contact pressure distribution (measurement).

Three variants were considered to analyze the multi-level, cross-scale performance of the biomimetic adhesive shoes. The biomimetic adhesive shoe with a buffering layer and a biomimetic adhesive layer is designated as BA-S (buffer and adhesion shoes). The control group, comprising a shoe with an adhesive layer without a buffering layer, is labeled as N-B-S (no-buffer and adhesion shoes). The N-B-A (no-adhesion and buffer shoes) group refers to the biomimetic adhesive shoe with a buffering layer but without an adhesive layer (Figure 4a). The experiment was conducted with a constant impact speed of 50 cm/s, a preload of 70 N, and an impact applied to the aluminum plate substrate connected to sensors. The buffering time for the N-B-S group is approximately 2 s, compared to the BA-S and N-B-A groups (~3 s) (Figure 4b). This reduced buffering time in the N-B-S configuration results from the absence of a buffering layer, which shortens the compression phase during impact and accelerates step completion.

Comparing the BA-S and N-B-S groups, evidently, the lack of biomimetic microstructures in the N-B-S group results in lower adhesion force and shorter adhesion time than in the BA-S group. Force analysis during compression and tension of the biomimetic adhesive shoe (Figure 4b inset) shows that during compression, pressure forces are the primary forces at play, concentrating the compression in the cushioning layer through force transmission.

In the microgravity environment, comparison of the compression behavior of BAS and N-B-S under the same stepping pressure (Figure 4c) shows that the maximum compression of BAS during a single step can reach 9.9 mm, which is significantly greater than that of N-B-S (2.2 mm). This indicates that the cushioning layer of the biomimetic adhesive shoes can effectively reduce shocks and impacts during stepping and walking. As illustrated in the inset of Figure 4c, during rapid descent and ground contact, the cushioning layer of BAS first undergoes compression, thereby prolonging the force application time, attenuating the impact, and subsequently recovering.

The maximum adhesion force for BA-S (75 N) is higher than that for N-B-A (50 N). This difference can be explained by the schematic in Figure 4d, which illustrates the detachment process from the edge of the biomimetic adhesive shoe. As the BA-S shoe features micropillars on its contact surface, cracks that form during detachment must overcome additional obstacles during their expansion. When encountering protrusions, the cracks are forced to bypass these protrusions, increasing the crack propagation path length and energy consumption. This phenomenon is described by Equation (4) [29]:

(4) $$\frac{F_{s}}{F_{c}}=\frac{E_{s}I_{s}w_{s}}{E_{c}I_{c}w_{c}}$$

where $\frac{F_{s}}{F_{c}}$ denotes the adhesion enhancement factor, *E_s_* is the elastic modulus of the stiff region, *I_s_* is the area moment of inertia of the stiff region, and *w_s_* is the actual contact width of the stiff region. Similarly, *E_c_*, *I_c_*, and *w_c_* represent the elastic modulus, area moment of inertia, and actual contact width of the compliant region, respectively.

The relationship reveals that increasing the contact width of the compliant region (*w_c_*) effectively enhances the overall adhesion performance. Conversely, the N-B-S group lacks surface microstructures and presents a relatively smooth contact interface, enabling cracks to propagate more easily, thereby reducing adhesion strength.

Furthermore, protrusions on the contact surface induce stress concentration under loading. These stress concentration zones hinder crack propagation because greater energy is required for a crack to continue growing through these localized high-stress regions. This effect is particularly pronounced on surfaces with microstructural protrusions, improving overall adhesion strength due to increased crack resistance.

Figure 4e illustrates the relationship between the adhesion force of biomimetic adhesive shoes and the number of steps under microgravity conditions. The experiment was conducted at a stepping frequency of 30 steps/min on an aluminum alloy substrate. As shown in Figure 4e, the adhesion force of BAS decreased by approximately 12.5% within the first 90,000 steps, followed by a rapid decline thereafter. This degradation is attributed to the repeated action of normal preload, tensile force, and tangential friction, which damaged the micropillar structures of the adhesive layer, thereby reducing the intermolecular contact area between the adhesive layer and the substrate and ultimately lowering the adhesion force. The inset of Figure 4e illustrates the forces acting on BAS when stretched at an angle *α*, including the tensile force ($F_{n}$), ground reaction force (*F_vac_*), adhesive resistance force (*F_vdW_*), and tangential frictional force (*F_f_*). Mechanical analysis of the cushioning layer indicates that, during tensile detachment at angle *α*, tangential frictional and normal adhesion forces are mitigated by the buffering effect of the micropillars, which also facilitates uniform stress distribution and concentrates the load within the micropillars. Within a two-dimensional plane-strain framework consisting of a rigid loading plate, an elastic buffer layer, an effective adhesion/friction interface, and a rigid substrate, a normal preload was applied to establish contact, followed by a small rotational perturbation to realize angled peeling. The computed interfacial normal contact pressure $p_{n}$ and the von Mises stress field within the buffer layer conform to the colormap (0–400 kPa) in Figure 4e. With increasing peeling angle α, pressure magnitude and shear–tension coupling at the trailing edge are markedly amplified; the peak $p_{n}$ increases and migrates toward the trailing edge, indicating an edge-dominated detachment process. Concurrently, the buffer layer exhibits a through-thickness “stress-diffusion cone,” characterized by smoother iso-stress contours and a reduced peak-to-mean ratio, evidencing redistribution and diffusion of interfacial loads and the suppression of local stress concentrations. These trends suggest that a moderate buffer thickness promotes pressure homogenization and peak attenuation without compromising controllability, whereas an overly thin layer is insufficient for diffusion, and an overly thick layer introduces excessive compliance. The numerical results are consistent with the spatial distribution of the pressure scale in Figure 4e and provide a mechanistic rationale for the enhanced stability and comfort of the biomimetic adhesive footwear under angled loading α.

Under microgravity conditions, the biomimetic adhesive shoe demonstrates considerable application potential owing to its strong adhesive capabilities. One such application is in astronaut exercise, aiming to mitigate the risk of muscle atrophy. In this study, volunteers wore the biomimetic adhesive shoes while suspended by a constant-force suspension system to simulate microgravity, with EMG sensors attached to relevant lower limb muscle groups.

During leg-raising movements, the adhesion force provided by the biomimetic adhesive shoes simulated ground reaction forces to stimulate the muscular system. A control experiment was conducted with participants wearing shoes that lacked the biomimetic adhesive layer. An aluminum alloy plate with a surface roughness of Sa = 0.2 μm was used as the contact substrate. The stepping frequency was set to 30 steps/min, corresponding to a detachment speed of 75 cm/s for the adhesive shoe. The average detachment force recorded was approximately 80 N.

**Figure 5:**
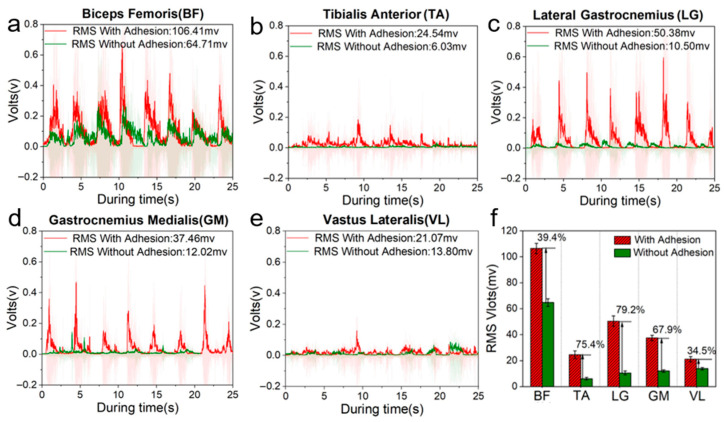
(**a**–**e**) Root mean squared results of the biceps femoris muscle, tibialis anterior, peroneal lateralis muscle, peroneal medialis, and vastus lateralis muscle in a microgravity environment, with the raw EMG data showing shades; (**f**) root mean squared average value of EMG leg lifting movements in subjects with (red) and without (green) biomimetic adhesive shoes in a microgravity environment.

Figure 5a–e show EMG signals and corresponding RMS values from the main force-generating muscle groups during stepping, both with and without the biomimetic adhesive shoes. The multiple peaks in each EMG trace indicate repeated activation of the respective muscle groups during the stepping cycles.

EMG signals (RMS) were consistently weaker when participants did not wear the adhesive shoes. The biceps femoris exhibited the highest RMS values among the recorded muscle groups, indicating its dominant role in leg lifting regardless of shoe condition. However, for muscles such as the tibialis anterior, peroneus longus, medial gastrocnemius, and vastus lateralis, EMG values were significantly higher when participants wore the biomimetic adhesive shoes, suggesting that detachment motions relied more heavily on calf muscle contractions. *An* engineering threshold was defined from the Δ*EMG%–F_n_* curve as the knee point of a piecewise linear fit with two lines; this knee point represents the minimum *F_n_* that yields a clear increase in Δ*EMG%*. As a stability cross-check, a ≥95% gait success rate (absence of premature detachment or major slip) was additionally required. Group-level recommendations derived from these knee points are provided in Dataset S2; per-subject thresholds with fit diagnostics and 95% confidence intervals are listed in Dataset S3; the paired raw cells used for estimation (Δ*EMG%* and *F_n_*) are provided in Dataset S4.

Figure 5f directly compares EMG amplitudes for each muscle group with and without the adhesive shoes. The results show significant increases in EMG signal amplitudes when using the biomimetic adhesive shoes, with improvements ranging from 34.5% to 79.2%. Statistical details (normality checks; paired *t*/Wilcoxon; one-/two-way ANOVA for shoe × speed/roughness; *p*-values, effect sizes, and 95% CIs) are summarized in Dataset S5. This demonstrates the effectiveness of the biomimetic adhesive shoes in stimulating lower limb muscle activity.

Compared to traditional exercise devices, these shoes stimulate key flexor groups such as the biceps femoris and gastrocnemius during microgravity stepping tasks, supporting their application in astronaut musculoskeletal conditioning.

## 4. Conclusions

Inspired by the omnidirectional mobility of climbing organisms, this study proposes a multi-level, variable-modulus, detachable biomimetic adhesive shoe. Experimental investigations under various detachment speeds, angles, and preload conditions demonstrate that the biomimetic adhesive shoe possesses low preload, strong adhesion, and detachable capabilities. During stepping exercises, the shoe generates plantar adhesion forces ranging from 50 to 105 N. The shoe comprises an upper layer, a cushioning layer, and an adhesive layer. The adhesive layer adapts to substrates with different curvatures, roughness, and materials, while the cushioning layer effectively mitigates impacts during the detachment process, extending the duration of the force and ensuring stable detachment.

Additionally, the EMG and heart rate data collected from participants wearing the biomimetic adhesive shoes show significant muscle stimulation, particularly in the biceps femoris and other flexor muscles. This research highlights the potential for developing a biomimetic adhesive shoe designed explicitly for flexor muscle training, offering new exercise methods and operational modes for astronauts.

## Data Availability

The main data that support the results in this study are available within the paper and the Supporting Information or are available for research purposes from the corresponding authors upon reasonable request.

## Supplementary Materials

The following supporting information can be downloaded at: https://www.mdpi.com/article/10.3390/biomimetics10120800/s1, Figure S1: Contact surface roughness maps and parameters measured by laser confocal microscopy across the tested Sa range (0.02–5 μm); Figure S2: Constant-tension suspension system for the simulated-microgravity trials—device photo and schematic; the mechanical-performance explanation is provided in Supplementary Methods S3.1, with statics and geometric derivations (Equations (S1)–(S4)); Figure S3: Electrode placement for lower-limb EMG (BF, TA, LG, GM, VL); Video S1: Demonstration of suspension-based microgravity stepping (BA/N-A/No-shoe conditions); Dataset S1 (CSV): Per-subject, per-condition paired observations for the EMG–contact-mechanics study (metadata, loads, EMG_RMS, Δ*EMG%*, trial indices); Dataset S2 (CSV): Group-level threshold recommendations from two-segment fits of ΔEMG% versus $F_{n}$ with the ≥95% gait-success engineering check; Dataset S3 (CSV): Per-subject thresholds (e.g., $F_{\mathrm{knee}}$ and stability-checked values) with fit diagnostics and 95% CIs; Dataset S4 (CSV): Raw paired cells used for threshold estimation (Δ*EMG%* and $F_{n}$ by subject/condition/level) including trial identifiers and covariates; Dataset S5 (CSV): Statistical summary for EMG and adhesion (normality tests; paired *t*/Wilcoxon; one-/two-way ANOVA for shoe × speed/roughness) with *p*-values, effect sizes (Cohen’s *d*, η^2^/partial-η^2^), and 95% confidence intervals.
