# Supplementary material for: Anti-Weightlessness Physiological Protection for the Lower Limb Muscle System Based on Biomimetic Adhesive Force Stimulation
Source: Biomimetics (Basel). 2025 Nov 28;10(12):800. doi: 10.3390/biomimetics10120800 (PMC12730586; doi:10.3390/biomimetics10120800)
Supplement: Supplementary file 1 [file biomimetics-10-00800-s001.zip › Supplementary MethodsS3.1.pdf]

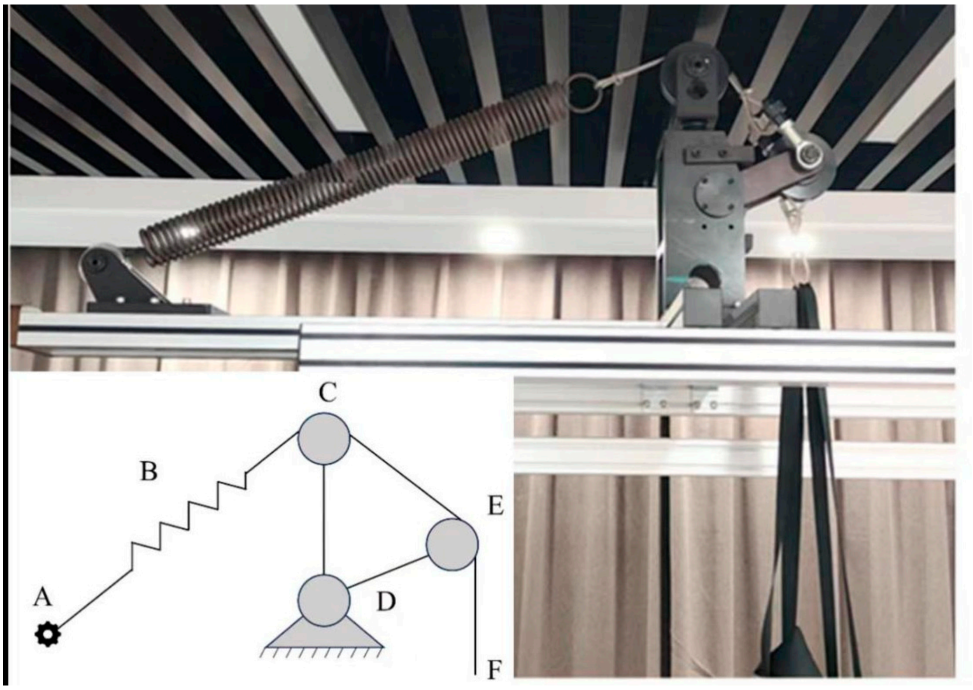

Figure S3 illustrates a constant-tension suspension system; panel shows a photograph of the device with an inset schematic of its mechanical layout.

In the inset of Figure S3, A denotes the adjusting nut that sets the spring preload; B the spring element; C and D the fixed pulleys; E the movable pulley; CD and DE the connecting rods; and CE and EF the steel cables.

A force equilibrium analysis yields the following expression:

$$kxsin\beta = F_t sin\alpha \quad (1)$$

where k is the spring stiffness coefficient, x is the spring's elongation, and F_t is the tension force exerted at point F. According to the sine rule, the geometric relationship among the components is:

$$\frac{CE}{sin\alpha} = \frac{CD}{sin\beta} \quad (2)$$

Substituting equation (2) into equation (1) leads to:

$$F_t = kx\frac{CD}{CE} \quad (3)$$

By selecting an appropriate length of the steel cable such that the spring remains unstretched when pulley E is in its initial position, the condition x = CE can be maintained throughout operation. Substituting this into equation (3) simplifies the tension expression to:

$$F_t = kCD \quad (4)$$

Thus, with properly chosen structural parameters, the system can reliably deliver a constant tension output.
