# Supplementary figures and images for: Anti-Weightlessness Physiological Protection for the Lower Limb Muscle System Based on Biomimetic Adhesive Force Stimulation
Source: Biomimetics (Basel). 2025 Nov 28;10(12):800. doi: 10.3390/biomimetics10120800 (PMC12730586; doi:10.3390/biomimetics10120800)

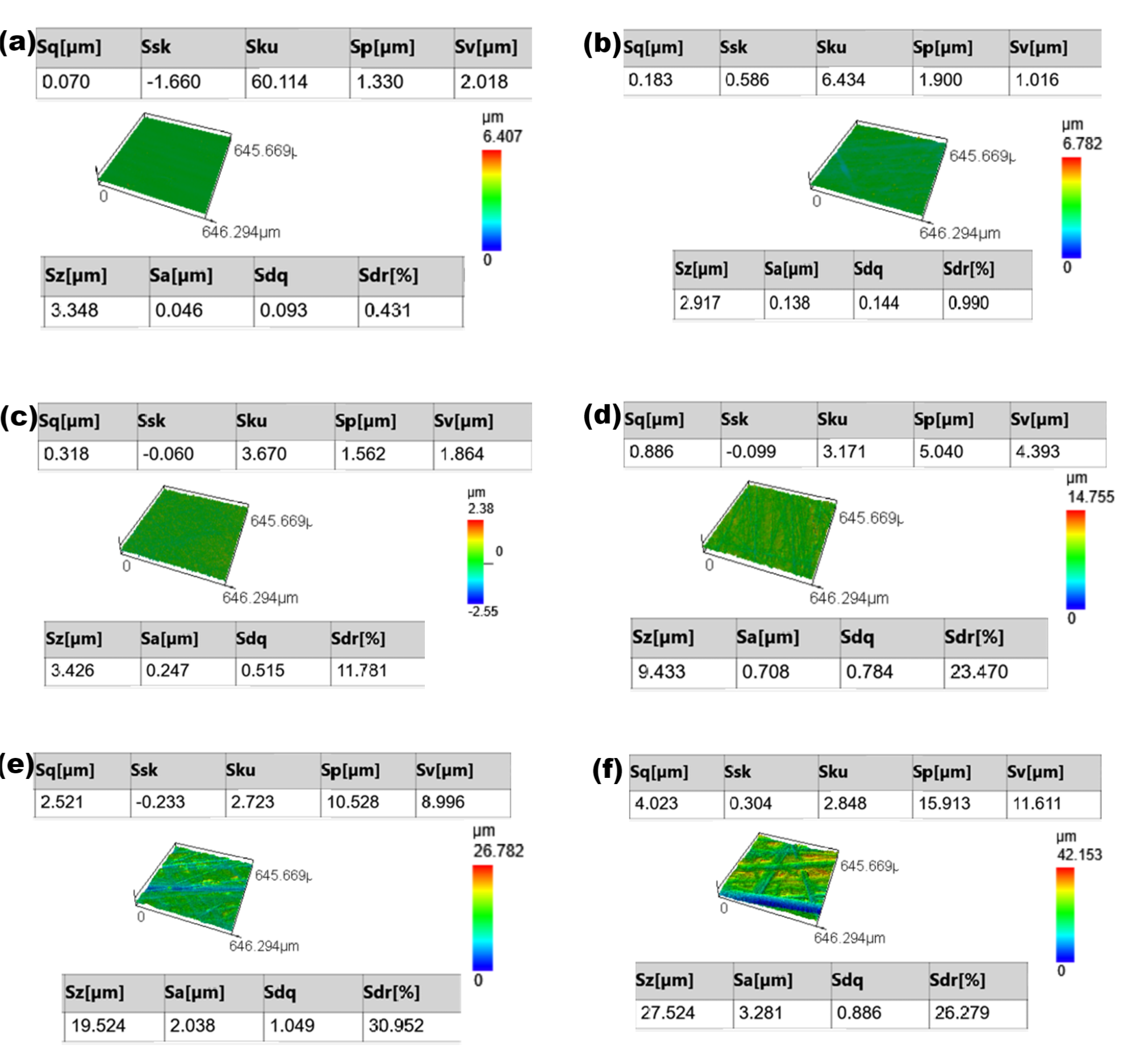

Supplement: Supplementary file 1 [file biomimetics-10-00800-s001.zip › FigureS1.png]

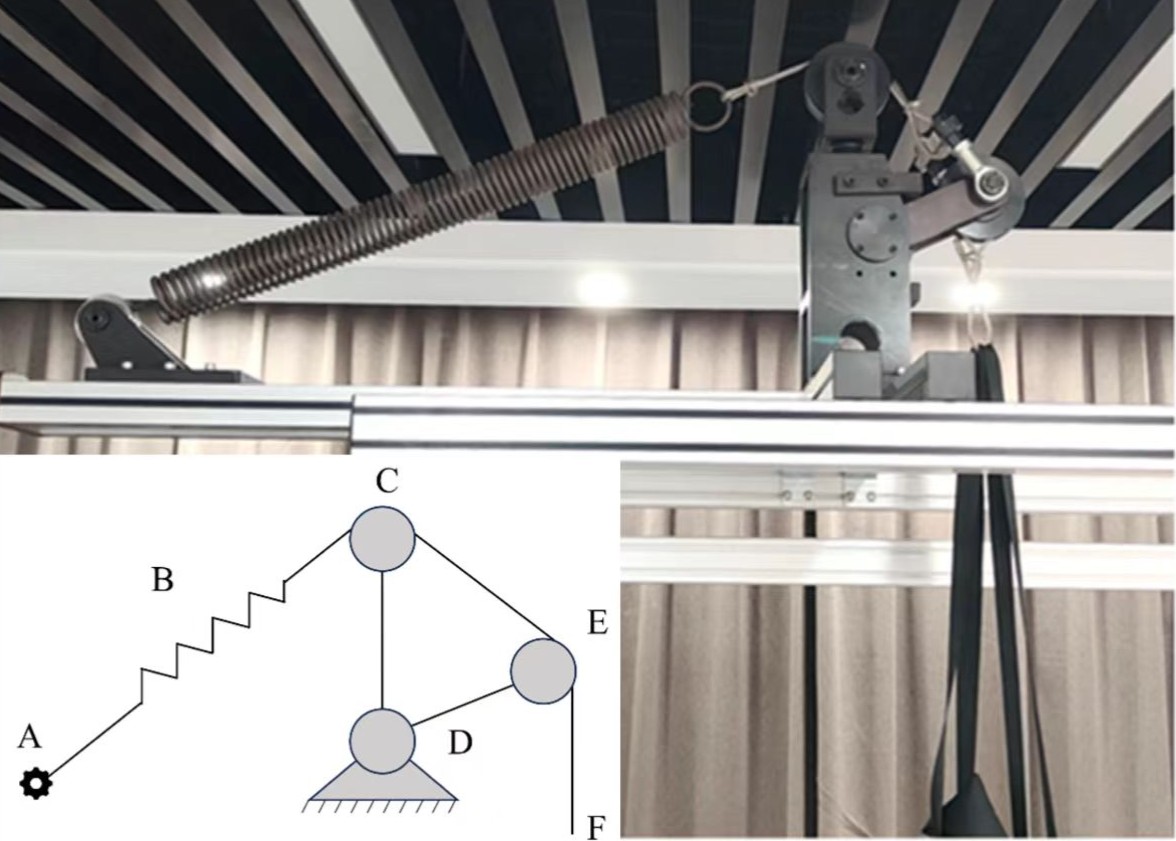

Supplement: Supplementary file 1 [file biomimetics-10-00800-s001.zip › FigureS2.jpg]

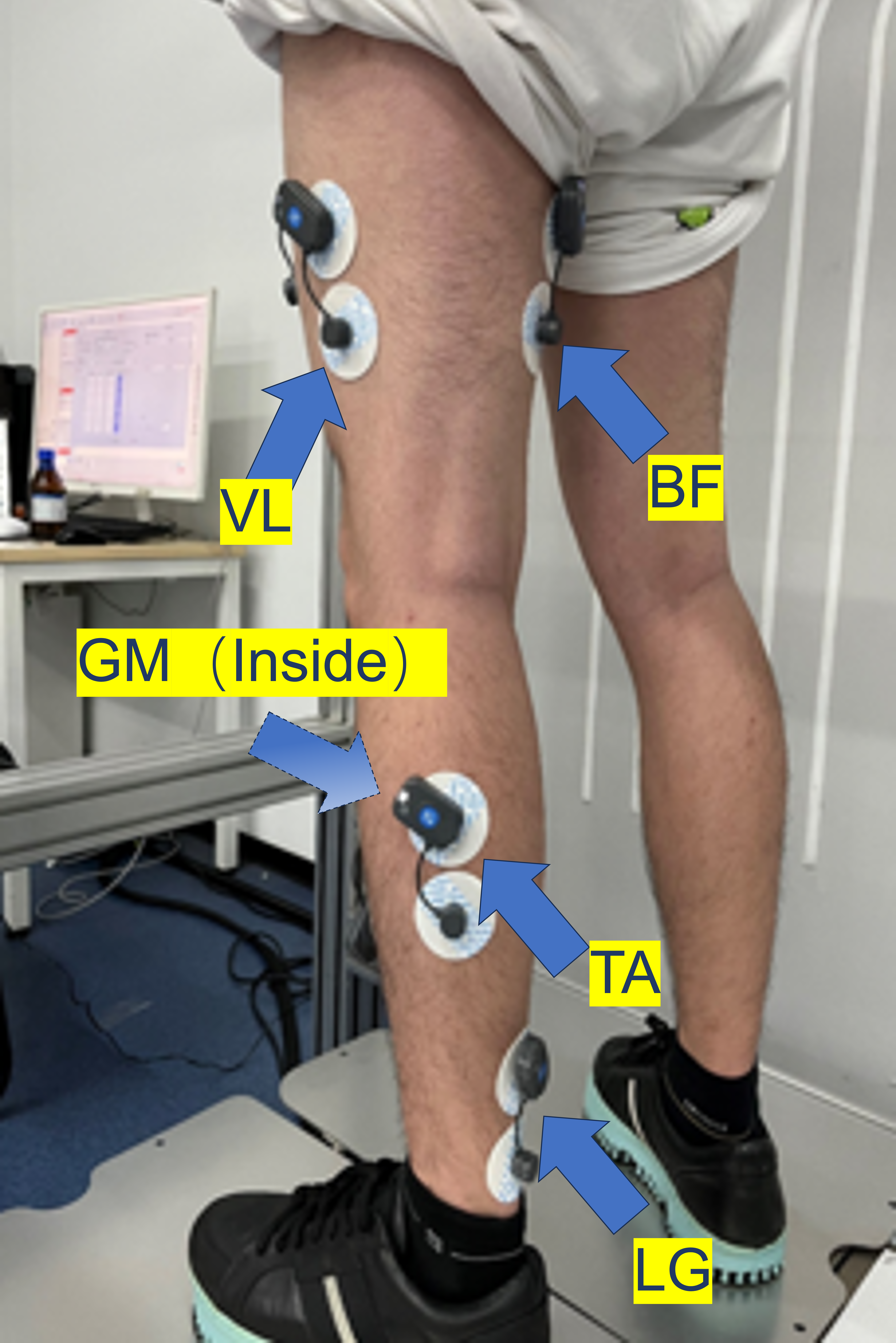

Supplement: Supplementary file 1 [file biomimetics-10-00800-s001.zip › FigureS3.png]
